# Immunotherapy-Induced Anterior Hypophysitis

**DOI:** 10.7759/cureus.16538

**Published:** 2021-07-21

**Authors:** Pranali S Pachika, Razwana Khanam, Seemal Faisal, Tahreem Ahmad, Anjana Chandrasekhara Pillai

**Affiliations:** 1 Internal Medicine, University of Pittsburgh Medical Center-McKeesport, Pittsburgh, USA; 2 Internal Medicine, University of Pittsburgh Medical Center-Mckeesport, Pittsburgh, USA

**Keywords:** pd-1, pd-l1, hypophysitis, checkpoint inhibitors, ipilimumab, nivolumab, immunotherapy-related adverse events, cancer-immunotherapy

## Abstract

Immunotherapy-based regimens are currently the standard treatment for many different types of cancers. Monoclonal antibodies against cytotoxic T lymphocytes antigen (CTLA-4), programmed cell death protein-1 (PD-1), and PD-1 ligand (PD-L1) are the major subgroups of immune checkpoint inhibitors, which are being used widely in the treatment of various malignancies. They function by reactivating the immune system against the tumor cells but can also trigger autoimmune side effects, which are termed immune-related adverse effects (irAEs). In this report, we present a case of irAEs in a patient treated for colorectal cancer with combination therapy with ipilimumab (anti-CTLA-4 antibody) and nivolumab (anti-PD-1 antibody).

## Introduction

Immune checkpoint inhibitors have evolved to be the standard treatment regimen for many cancer types. Monoclonal antibodies against cytotoxic T lymphocytes antigen (CTLA-4), programmed cell death protein-1 (PD-1), and programmed cell death ligand molecules (PD-L1) are the major subgroups of immune checkpoint inhibitors. They function by reactivating the immune system against the tumor cells; however, they can also trigger autoimmune side effects called immune-related adverse effects (irAEs). Endocrinopathies are the most noticed irAEs, and a few of these endocrine adverse events can be life-threatening, warranting a timely diagnosis and prompt management. Hypophysitis secondary to these drugs is one such side effect that requires timely diagnosis and management. Ipilimumab, a CTLA-4 inhibitor, has been shown to cause hypophysitis more frequently than other immunotherapy drugs, with a dose-dependent frequency ranging from 0% to 17.5% [1]. Combination therapies including both CTLA4 and PD1 or PDL1 inhibitors tend to increase the incidence of potential irAEs [2]. We describe the case of a patient who developed anterior hypophysitis after being treated with ipilimumab and nivolumab for advanced colorectal carcinoma.

## Case presentation

A 64-year-old Caucasian man with metastatic colorectal cancer recurrence being treated with ipilimumab and nivolumab presented with a persistent central throbbing headache for five days. He had undergone his first immunotherapy cycle one month prior to the onset of his current symptoms. He reported having a central and non-radiating headache, which was sharp and throbbing. When the pain had started, he had noted its intensity to be 4-5, but later it had progressed and had gotten worse, and at the time of admission, he mentioned the intensity of pain to be 9/10. His pain was associated with nausea and fatigue. He denied any fever, chills, vision changes, diplopia, weakness, numbness of the extremities, hearing loss, tinnitus, or gait changes.

**Figure 1:**
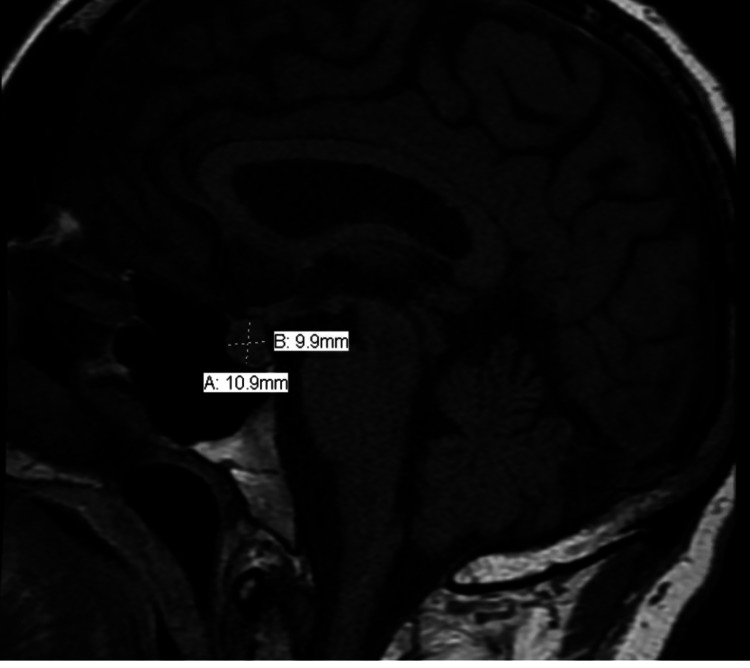
MRI brain showing pituitary gland enlargement in sagittal section MRI: magnetic resonance imaging

**Figure 2:**
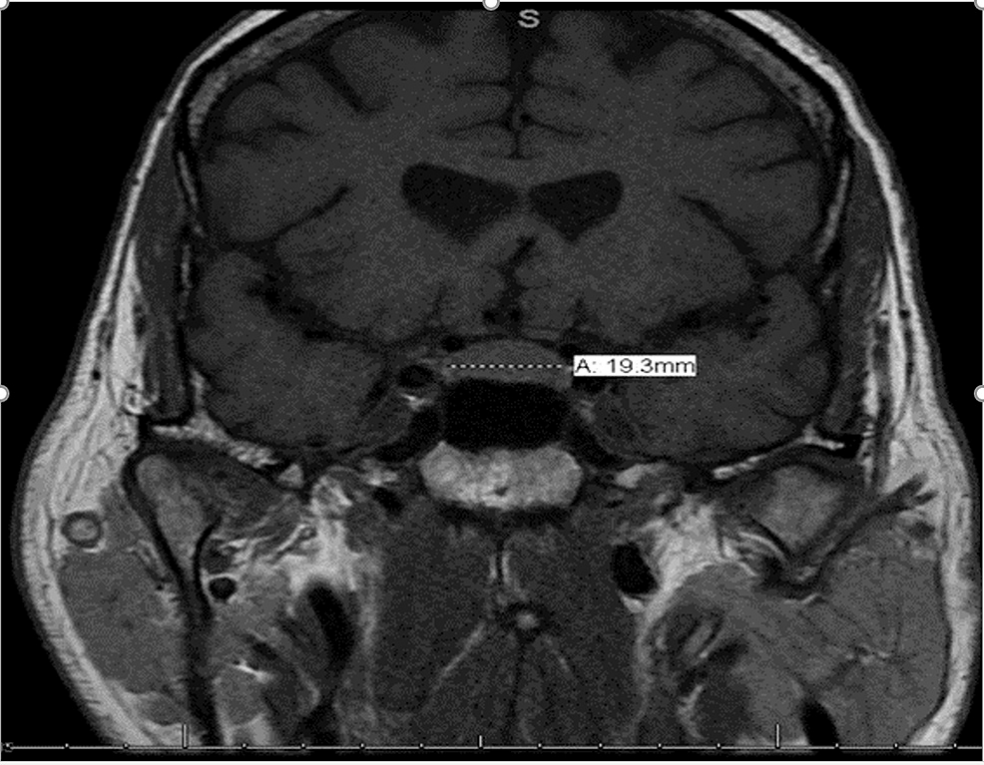
MRI brain showing pituitary gland enlargement in coronal section MRI: magnetic resonance imaging

His physical examination was unremarkable without any focal neurological deficits. Given his new-onset headache in the setting of immunotherapy, further investigations were conducted. CT head was unremarkable for any acute intracranial abnormalities and intracranial masses. MRI brain was performed, which showed mild prominence of the pituitary gland measuring about 11 mm in the craniocaudal dimension (Figures 1, 2). In the setting of his treatment with immunotherapy and MRI findings, a diagnosis of hypophysitis secondary to immunotherapy was considered and further workup was done. His hypophysitis was complicated by hypogonadism [luteinizing hormone (LH) level: 1 MIU/ml, testosterone level: 19 ng/dl], hypothyroidism [thyroid-stimulating hormone (TSH) level: 0.18 uIU/ml, FT4 level: 0.8 ng/dl], and adrenal insufficiency [adrenocorticotropic hormone (ACTH) level: <5 pg/ml, cortisol level: 0.7 mcg/dl]. The patient was first started immediately on stress dose IV hydrocortisone, which significantly improved his symptoms, and he was later started on levothyroxine. To avoid an adrenal crisis, steroids were administered before levothyroxine. Steroids were gradually tapered. On day one, he received 50 mg IV hydrocortisone every six hours, followed by 50 mg every eight hours on the second day, and 50 mg every 12 hours on the third day. He was put on oral steroids since day four, taking 40 mg of hydrocortisone in the morning and 20 mg in the evening. Oral steroids were gradually tapered as well, and he was discharged with 20 mg of hydrocortisone in the morning and 10 mg in the evening. Post-discharge, he closely followed up with the endocrinology and oncology. Weighing the risks versus benefits, he was rechallenged with nivolumab monotherapy with close outpatient follow-ups. Currently, he remains asymptomatic; however, he still continues to be on oral steroids (20 mg/10 mg) for low cortisol levels.

## Discussion

Immunotherapy in the form of checkpoint inhibitors is one of the prominent advancements in the field of Immuno-oncology and it has made significant strides in the availability of treatment options for many advanced malignancies. These drugs have been approved by the FDA for the treatment of a wide variety of advanced malignancies. Even though they have been known to have fewer side effects and are well tolerated by the patients compared to traditional chemotherapy, they are associated with a set of unique side effects called irAEs [3]. The role of cytotoxic T lymphocytes in tumor suppression is similar to their role in the infection. Tumor cells express some surface receptors that the normal cells do not express, which will be identified by our immune system and activate the cytotoxic T lymphocytes. Activated T lymphocytes destroy the tumor cells and inhibit cancer development and progression. Monoclonal antibodies against CTLA-4, PD-1, and PD-L1 molecules are the major subgroups of immune checkpoint inhibitors.

PD-1 and PD-L1 are both transmembrane proteins. PD-1 is expressed on the T cells, natural killer (NK) cells, and macrophages [4]. PD-L1 is expressed on the surface of many tissues including the surface of the tumor cells. PD-L1 expressed by the tumor cells helps in developing an escape mechanism from the immune system. PD-L1 expressed on the tumor cells binds to the PD-1 on the surface of the activated T cells and inhibits the secretion of the cytokines, thereby preventing apoptosis leading to the uninhibited growth of the tumor cells. Monoclonal antibodies developed against the PD-1 and PD-L1 receptors bind to them, inhibiting the interaction between these receptors, preventing the deactivation of the cytotoxic T cells, and thereby suppressing the tumor growth [5]. Nivolumab is one of the monoclonal antibodies against the PD-1 receptor, which has been approved for the treatment of colorectal cancer [6]. CTLA-4 and CD28 are the receptors present on the surface of the T cell, which bind to the B7 molecules on the antigen-presenting cells and play a key role in the activation of the T cells. CTLA-4 has a higher affinity than CD28 to B7 molecules. When attached to the B7 molecule, CD28 helps in the activation of the T cell, while CTLA-4 helps in the downregulation of the T cell as well as in maintaining a balance in the immune activation. So, blocking the interaction between the CTLA-4 and B7 molecule using a monoclonal antibody against CTLA-4 helps in the activation and proliferation of the T cells and cytokine release, which will help in destroying the tumor cells and cancer progression [7]. Ipilimumab is an anti-CTLA-4 antibody approved for cancer therapy. Thus, using anti-PD-1, PD-L1, and CTLA-4 antibodies results in the activation of the immune system and leads to the rare side effects called irAEs. Side effects and toxicities due to immunotherapy have been reported in various organ systems. Endocrine-related adverse side effects are commonly reported irAEs. A meta-analysis of clinical trials of various immune checkpoint inhibitors has been done to evaluate the incidence of the various endocrinological adverse events with either a single agent or combination therapy. The highest rate of Incidence of the hypophysitis was observed in the combination therapy with CTLA-4 inhibitor and either PD-1 or PD-L1 inhibitors, at 6.4%; it was 3.2% with CTLA-4 inhibitors, 0.4% with PD-1 inhibitors, and was least in PD-L1 inhibitors with less than 0.1% [8].

Hypophysitis is the inflammation of the pituitary gland resulting in the decreased production of the hormones. If not recognized and treated promptly, it can be life-threatening. Given its high incidence associated with the combination therapy, awareness regarding this possible side effect is critical. The mechanism of hypophysitis remains unclear but the possible hypothesis is the T cell-mediated immune destruction [9]. Patients can have a variety of symptoms including headaches, vision changes, or those consistent with other endocrine abnormalities due to the secondary deficiency of the hormones. Such a presentation should prompt immediate workup with brain imaging and hormonal blood work. Characteristic findings seen on the MRI brain are hypo-enhancing lesions in the anterior lobe of the pituitary gland suggesting fibrosis of the gland [10]. Depending on the severity of the patient's symptoms, the management includes initiation of corticosteroid therapy, hormone replacement therapy to prevent patients from going into adrenal crisis, and sometimes even discontinuation of the offending agent either temporarily or permanently [11].

## Conclusions

Given the advances in cancer treatment with immunotherapy and the fact that it has now become a mainstream therapy for many cancers, physicians need to be aware of its most widely recognized side effects. Our case report discussed one such important immune-related adverse effect in a patient treated for colorectal cancer.
